# Risk of faecal pollution among waste handlers in a resource-deprived coastal peri-urban settlement in Southern Ghana

**DOI:** 10.1371/journal.pone.0239587

**Published:** 2020-10-02

**Authors:** James-Paul Kretchy, Mawuli Dzodzomenyo, Irene Ayi, Duah Dwomoh, Kofi Agyabeng, Flemming Konradsen, Anders Dalsgaard

**Affiliations:** 1 Department of Physician Assistantship Studies, School of Medicine and Health Sciences, Central University, Miotso, Accra, Ghana; 2 Department of Biological, Environmental and Occupational Health Sciences, School of Public Health, College of Health Sciences, University of Ghana, Legon, Accra, Ghana; 3 Department of Parasitology, Noguchi Memorial Institute for Medical Research, College of Health Sciences, University of Ghana, Legon, Accra, Ghana; 4 Department of Biostatistics, School of Public Health, College of Health Sciences, University of Ghana, Legon, Accra, Ghana; 5 Department of Public Health, Faculty of Health and Medical Sciences, University of Copenhagen, Copenhagen, Denmark; 6 Department of Veterinary and Animal Sciences, Faculty of Health and Medical Sciences, University of Copenhagen, Copenhagen, Denmark; 7 School of Chemical and Biomedical Engineering, Nanyang Technological University, Singapore; University of Brescia, ITALY

## Abstract

Resource-deprived coastal peri-urban settlements in Southern Ghana are characterized by indiscriminate solid waste disposal and open defecation practices. Persons engaged in waste handling in such communities perform their activities with little or no personal protective equipment. They are thus confronted with the risk of faecal pollution of the hands and other bodily parts. A mixed method approach was used to investigate 280 waste handlers performing different activities to estimate recent faecal pollution of their hands and to observe the utilization of personal protective equipment and sanitation/hygiene facilities during work. The log concentration of *E*. *coli* on hands of waste handlers after work (8.60 ± 4.20 CFU/hand, mean ± standard deviation) was significantly higher compared with the *E*. *coli* log concentration before work (2.95 ± 1.89 CFU/hand, mean ± standard deviation) (p<0.001). The odds of faecal pollution was significantly higher (aOR 4.2; 95% CI: 1.9–9.1) for workers aged 35 years and above compared with those less than 35 years; and for workers at public toilet facilities (aOR 3.0; 95% CI: 1.0–8.4) compared with those who worked for private waste handling companies. Female workers were, however, 60% less likely (aOR 0.4; 95% CI: 0.2–0.8) to experience faecal pollution of their hands compared with males. The workers had limited access to water and sanitation and hygiene facilities, and about one-fifth (n = 59; 21.1%) did not use personal protective equipment during work. Waste handlers should be provided and instructed in proper use of personal protective equipment, have access to sanitation facilities and adopt improved hygiene behaviour to avoid the risk of faecal pollution and associated disease risk.

## Background

The world is faced with increasing challenges regarding the provision of sustainable sanitation and waste management. These challenges impact negatively on both the environment and health of persons exposed to faecal-contaminated solid waste. In many resource-deprived peri-urban settlements in low- and middle-income countries (LMICs) like Ghana, these problems have worsened over the years due to rapid urbanisation and inadequate waste management and sanitation infrastructure [1, 2]. The evidence of the weak sanitation and waste management facilities in these settings have been described in various studies, such as overflowing garbage containers, indiscriminate waste disposal, overcrowded toilet facilities, choked open drains, defecation in open spaces and a general sense of unaesthetic environment [1, 2]. The solid waste generated might be polluted by straying animal faeces or human faeces from open defecation at the same sites that solid waste is disposed. It may also be from child faeces or diapers being disposed with solid waste or faeces deposited in plastic bags (so called “flying toilets”) and thereby exposing both inhabitants and waste handlers to faecal pollution [3].

Waste handlers working in areas of peri-urban communities, such as public toilet facilities, dump sites or open areas, who directly handle solid waste along the waste management chain, might experience high exposures to the various types and sources of faecal pollution, especially where the waste management systems are not sufficiently developed [3, 4]. The lack of or inadequate provision of personal protective equipment (PPE) and hygiene facilities by both public and private organisations employing a growing number of waste handlers worsen the risk factors of faecal exposures and associated health problems [5]. A review of the literature from many parts of the world including Asia [6], Europe [7], America [8] and Africa [9] indicate that waste handlers perform varied activities in peri-urban areas including sweeping, collection, transportation and disposal to reduce the large volumes of accumulated solid waste pile-ups whilst improving the general aesthetics of the environment. Risk factors such as the use of inappropriate PPE, lack of access to sanitation and hygiene facilities as well as poor hygiene behaviour by many waste handlers during working hours may compromise their health and safety. Waste handlers are repeatedly exposed to various pathogens whilst working in faecal polluted environments, either through direct physical contact with solid waste or by the inhalation of air-borne particulate matter [10, 11]. These exposures may be associated with hygiene-related diseases particularly diarrhoea and respiratory diseases [12, 13].

The concentration of the faecal indicator *Escherichia coli* (*E*. *coli*) on hands of persons has been used to estimate recent exposures to human and animal faeces and as a proxy to predict risk levels for the transmission of sanitation and hygiene-related pathogens. Even though *E*. *coli* has been used to assess exposures to faecal pollution among mothers, health-care workers and food handlers in different parts of Africa and Europe [14–17], there still remains paucity of information regarding the degree of faecal pollution among waste handlers in resource-deprived peri-urban communities in LMICs. The objective of this study was therefore to assess the level of faecal pollution of hands and associated risk factors among solid waste handlers in a coastal peri-urban community in Southern Ghana. Recommendations are also provided on how health risks can be prevented and controlled among the waste handlers.

## Methods

### Description of waste handlers and waste

Waste handlers were defined as sweepers, collectors, transporters and disposers, or those performing multiple waste handling activities i.e. sweeping and collection, collection and disposal or sweeping, collection and disposal who directly handled waste materials along the waste management chain. A detailed description of waste handler activities has been provided in an earlier publication by our group [5]. The type of waste managed included solid waste generated from households, mixed with fresh and decomposed human and animal faeces or effluents from domestic waste pipes and septic sludge from tanks emptied into open drains.

### Study area

The study was conducted in the coastal peri-urban community of Prampram, Ghana. Prampram was the largest peri-urban settlement within the erstwhile Dangme-West district of Ghana. Currently Prampram is the administrative capital of the newly created Ningo-Prampram district located in the Southern part of Ghana. The district has a population of about 122,836 (GSS, 2013) and lies between latitude 5° 45’ South and 6° 05’ North and Longitude 0° 05’ East and 0° 20’ West.

The Prampram township has two major demarcations: Lower community (Lower East and Lower West), and Upper community (Kley and Olowe), where residents are occupied predominantly in fishing and subsistence farming. These communities were deprived of many sanitation and waste management infrastructure, such as toilet facilities and proper solid waste disposal sites. Over 50% of residents are engaged in open defecation practices, creating multiple environmental and health concerns associated with the faecal pollution of the solid waste stream [18].

### Recruitment of waste handlers

Stakeholders supervising waste handling activities in the Ningo-Prampram district were recruited from Dodowa in the erstwhile Dangme-West and the current Ningo-Prampram districts whilst the waste handlers were recruited from the Ningo-Prampram district. Stakeholder interviews with eight heads/coordinators of institutions supervising waste handling activities in the Ningo-Prampram district were first conducted to establish a platform for the identification and enumeration of waste handlers. The stakeholders were made up of district Environmental and Sanitation Health Officers (n = 2), Local Assembly Members (n = 4), District Waste Management Planning Officer (n = 1) and Coordinator of the Local Area Council for public toilet managers and waste transporters (n = 1) in Prampram. The objective of the interview was to identify waste handlers and describe specific types of activities performed. After doing a transect walk and observation of the study area to identify specific waste handling activities occurring at different sites (i.e. beaches, open dumping fields, public toilet facilities) and to enumerate waste handlers, 280 out of the total estimated 300 waste handlers gave informed consent and were therefore recruited to participate in the study. Convenient sampling was used to recruit the waste handlers.

### Data collection

Different methods and tools were used for data collection. Data were collected in four different ways; transect walk and observation, questionnaire, hand-rinse collection (before and after work) and laboratory microbial analysis. Data were triangulated to complement each other to provide information on sanitation and hygiene, surfaces exposed to waste, utilization of PPE as well as on faecal pollution of hands of waste handlers [19, 20].

#### Transect walk and observation

The transect walk begun at the outskirts of Prampram and gradually narrowed to the centre of the township, to ensure that all activities of interest were captured [21]. The starting point was determined after a brief interaction with stakeholders supervising waste handling activities in the area, who indicated that most of the solid waste pile-ups, public toilet facilities as well as open defecation practices at the beaches/bushes were found at the outskirts of the town. It was therefore easier to find waste handlers especially disposers, collectors and transporters at the outskirts, whilst sweepers were found when the transect walk was narrowed towards the centre of the Prampram township. The purpose of the transect walk and observations was to get familiarized with the study area and obtain information about the state of waste management, access to hygiene and sanitation facilities, as well as use of PPE among the waste handlers.

#### Questionnaire

A researcher-administered questionnaire comprising twenty items was used to obtain information from all 280 waste handlers which included their socio-demographic characteristics, types of activities performed, exposure of bodily surfaces as well as utilization of PPE. Questions were asked before starting a day’s work at locations where waste handlings took place (i.e. beaches, open dumping fields, public toilet facilities). Each respondent spent between 25–30 minutes to answer the itemized questions. The questionnaire was developed by reading the literature from Mbeng *et al*., (2009) and Ifegbesan (2010) [22, 23] and modified within the context of the current study to reflect, for example, the different age range and average monthly salary of the waste handlers. The questionnaire was administered to the workers after a pre-test was carried out at Dodowa, a nearby peri-urban setting with similar features as Prampram. The questionnaire was interpreted to waste handlers who could not read and write, by trained bi-lingual speaking research assistants.

#### Collection of hand rinse samples

After administering the questionnaire, two hand-rinse samples were collected from all 280 waste handlers, i.e. before and after engaging in a day’s work. Hand-rinse before work was collected before initiation of work, between 5:30 am to 6:00 am when workers reported to work, whilst that for after work was collected between five to ten minutes after work was completed, averagely between 9:00 am to 9: 30 am. The right hand (comprising finger tips, nails, palm and back of the hand, up to the wrist level) of each waste handler was placed in a sterile sampling bag [24, 25]. Fifty ml sterile distilled water [25, 26] was poured from Falcon tubes onto the hands in the bag which were gently massaged for about 60 seconds [27] to obtain hand-rinse of the microbiological flora of the hand. The hand-rinse was poured back carefully into the Falcon tubes. The hand was then wiped with tissue paper. The Falcon tubes containing the hand-rinse samples were placed in a cold box with ice packs which maintained the box at temperature ≤ 10°C [28] and transported (between 45 minutes and 1 hour) to the laboratory of the Noguchi Memorial Institute for Medical Research for culture and enumeration of *E*. *coli*. The time between the collection and addition of hand-rinse samples onto agar plates at the NMIMR was approximately 4 hours 30 minutes for before work and 2 hours 30 minutes for the after work samples.

#### Culture and enumeration of E. coli

The enumeration of *E*. *coli* in hand-rinse from the waste handlers was performed according to the methods outlined by Warburton, (2000) [29] with slight modifications as follows: 1 ml of hand-rinse water including from appropriate ten-fold serial dilutions was analysed by the pour plate method dispersing it into Petri-dishes which were then added and mixed with 20 to 25 ml of molten Brilliance^TM^ *E*. *coli* selective chromogenic indicator agar (CM 1046, Oxoid, UK) with a temperature of about 45°C. The agar plates were incubated at 44°C ± 0.5°C for 24 hours. Typical purple *E*. *coli* colonies were enumerated using a colony counter (Gallenkamp, UK). Where the colonies were too numerous to count, a 10^−1^ serial dilution was inoculated the next day to grow and enumerate *E*. *coli* colonies.

### Ethical considerations

The study protocols were approved by the Institutional Review Board of the Dodowa Health Research Centre, Ghana Health Service with review number (DHRC-IRB–STUDY NO.01/10/11) and the Ghana Health Service Ethical Review Committee (GHS-ERC– 09/07/12) before commencement of data collection. An informed consent form, explaining the objectives of the study, risks and benefits, right to refuse and confidentiality was given and explained to the participants. Those who agreed were voluntarily recruited, after receiving their written consents. Study participants were assured of confidentiality and anonymity. The waste management organisations with which the participants worked were anonymised. Each participant was treated with respect.

### Quality control

About 10% of the isolated colonies were purified and confirmed as *E*. *coli* using phenotypic tests including citrate production (Simmon Citrate Agar–CM 0155, Oxoid, UK), acid and gas production (Triple Sugar Iron slant agar–CM 0277, Oxoid, UK), indole production, motility (Sulphur Indole Motility media–CM 0435, Oxoid, UK) and urea production (Urea Agar–CM 0053, Oxoid, UK). The sterile distilled water was plated and used as negative controls. Duplicate plates of the hand-rinse samples were inoculated and colonies enumerated. Where the *E*. *coli* colonies were too numerous to count, a 10^−1^ serial dilution was inoculated the next day to grow and enumerate them. The mean counts of *E*. *coli* for each duplicate plate were used in the analysis. Stakeholder interviews were transcribed verbatim and transcripts were checked by the author for completeness and accuracy. The questionnaire was validated through a pre-test in Dodowa, a similar study setting as Ningo-Prampram.

### Data management and analysis

The observations from transect walk were systematically recorded using observational guides and into field notes. The data from questionnaire and *E*. *coli* enumerations were coded and entered into SPSS 17.0 for Windows 7 (SPSS, Inc., Chicago, IL.) and later imported into STATA MP Version 13 (STATA Corporation, College Station, USA) for statistical analysis. Continuous variables were summarized with means and standard deviations, while frequencies and percentages were reported for categorical variables. We used 50 ml of distilled water to rinse the right hand of each worker. From the hand-rinse water, one ml was analysed for *E*. *coli*. Thus, the limit of detection of *E*. *coli* was ≥ 50 CFU per hand. Therefore the concentration of *E*. *coli* per hand was computed by multiplying the number of colonies on each plate by a factor of 50 [30]. The difference in log concentration of *E*. *coli* before and after work was computed by subtracting the concentration before work from that of after work. The Welch t-test was used in comparing the average difference in log concentration of *E*. *coli* (i.e. after work minus before work) between two groups. The ANOVA was used to compare the average difference in log concentration of *E*. *coli* across three or more groups while Bonferonni test were used as a post-hoc test for pairwise comparison after the ANOVA test. When no *E*. *coli* were detected on culture plates of the hand-rinse sample, the results were set to 0.25 CFU/hand, because the logarithm of zero is undefined. The detection of *E*. *coli* was dichotomized into “detection” (visible growth of *E*. *coli*) and “no detection” (no visible growth of *E*. *coli*). Binary logistic regression model was performed to establish associations between risk factors and the detection of *E*. *coli* on hands. The statistical level of significance was set at 5%.

## Results

The transect walks and observations of waste management practices and general sanitary conditions revealed that waste handlers worked at different faecal polluted open spaces including the beaches, around ponds and refuse containers, public toilet facilities, cemeteries and in open drains receiving faecal sludge and domestic waste effluents. The researcher observed, from the transect walks, actual open defecation practices by residents on the beaches or in bushes as well as physical presence of human and animal excrements in the solid waste managed by the waste handlers. The workers were engaged in sweeping, collection, transportation and disposal of wastes as well as a combination of two or more activities (also known as multiple tasking). An estimated 95% of the workers had limited access to water, sanitation and hygiene facilities during waste handling, based on the researcher’s observations during transect walk. For example, workers had to wash their hands with self-purchased sachet water without soap, before eating or after defecation during a working day. The results of the observation from transect walk of the peri-urban community also showed that waste handlers used rudimentary waste handling equipment and technology such as brooms, wheel barrows and shovels. Waste handlers were observed working in hot and humid conditions, thus, non-compliance to the use of PPE may be linked to the discomfort in its use during a hot workday.

### Socio-demographic characteristics, distribution of waste handling practices and use of personal protective equipment

The socio-demographic characteristics of waste handlers performing different waste handling activities are shown in Table 1. A total of 280 waste handlers were recruited from five different waste management organizations operating within the study area with most of them being females (n = 211; 75.4%). The average age of the participants was 42.7 ± 12.8 years. A total of 115 workers (41.1%) had no formal education whilst only 13 workers (4.6%) had secondary education. A majority of waste handlers (n = 232; 82.9%) earned a monthly income of between 80–150 (GH₵) (approx. USD 36–45), far below the average per capita monthly income of Ghana of 225 (GH₵) (approx. USD 117) (GSS, 2013), which was the main source of earning for most waste handlers.

**Table 1:** Socio-demographic characteristics and type of personal protective equipment (PPE) by waste handlers performing different activities.

Socio-demographic characteristics/type of PPE	Waste handling activities n (%)								Total
	Sweeping (n = 51)	Disposal (n = 18)	Collection (n = 12)	Transport (n = 5)	Sweeping & Disposal (n = 6)	Sweeping & Collection (n = 69)	Collection & Disposal (n = 36)	Sweeping, Collection & Disposal (n = 83)	(N = 280)
Sex									
Male	3 (4.4)	12 (17.4)	8 (11.6)	5 (7.3)	2 (2.9)	3 (4.4)	29 (42.0)	7 (10.1)	69 (24.6)
Female	48 (22.8)	6 (2.8)	4 (1.9)	0 (0.0)	4(1.9)	66(31.3)	7 (3.3)	76 (36.0)	211 (75.4)
Age in years									
<35	15 (16.7)	6 (6.7)	4 (4.4)	1 (1.1)	0 (0.0)	26 (28.9)	10 (11.1)	28 (31.1)	90 (32.1)
35 and above	36 (19.0)	12 (6.3)	8 (4.2)	4 (2.1)	6 (3.2)	43 (22.6)	26 (13.4)	55 (29.0)	190 (67.9)
Highest Education Level									
None	25 (21.7)	7 (6.1)	3 (2.6)	1 (0.9)	3 (2.6)	26 (22.6)	14 (12.2)	36 (31.3)	115 (46.0)
Primary	11 (14.1)	4 (5.1)	4 (5.1)	0 (0.0)	0 (0.0)	28 (35.9)	10 (12.8)	21 (26.9)	78 (27.9)
JHS	7 (23.3)	3 (10.0)	1 (3.3)	0 (0.0)	1 (3.3)	5 (16.7)	4 (13.3)	9 (30.0)	30 (10.7)
MSLC	6 (13.6)	3 (6.8)	3 (6.8)	3 (6.8)	2 (4.6)	8 (18.2)	5 (11.4)	14 (31.8)	44 (15.7)
Secondary	2 (15.4)	1 (8.0)	1 (7.7)	1 (7.7)	0 (0.0)	2 (15.4)	3 (23.1)	3 (23.1)	13 (4.6)
No. of Years worked									
< 1 year	16 (9.9)	7 (4.3)	9 (5.6)	1 (0.6)	2 (1.2)	54 (33.3)	22 (13.6)	51 (31.5)	162 (57.9)
1–2 years	17 (53.1)	3 (9.4)	0 (0.0)	1 (3.1)	0 (0.0)	2 (6.3)	1 (3.1)	8 (25.0)	32 (11.4)
3–4 years	17 (22.1)	7 (9.1)	3 (3.9)	2 (2.6)	4 (5.2)	11 (14.3)	12 (15.6)	21 (27.3)	77 (27.5)
5 or more years	1 (11.1)	1 (11.1)	0 (0.0)	1 (11.1)	0 (0.0)	2 (22.2)	1 (11.1)	3 (33.3)	9 (3.2)
Current monthly salary									
Less than ¢80.0 (< USD 36)	14 (32.6)	7 (16.3)	0 (0.0)	1 (2.3)	3 (7.0)	5 (11.6)	2 (4.7)	11 (25.6)	43 (15.4)
¢80-¢150 (USD 36–45)	37 (15.6)	11 (4.6)	12 (5.1)	4 (1.7)	3 (1.3)	64 (27.0)	34 (14.4)	72 (30.4)	237 (84.6)
Type of personal protective equipment (multiple responses)									
Wellington boot (yes)	16 (9.3)	9 (5.2)	9 (5.2)	2 (1.2)	2 (1.2)	51 (29.5)	27 (15.6)	57 (33.0)	173 (61.8)
Gloves (yes)	25 (15.5)	9 (6.0)	11 (6.8)	0 (0.0)	2 (1.2)	39 (24.2)	24 (14.9)	51 (31.7)	161 (57.5)
Mouth/nose cover (yes)	15 (16.7)	5 (5.6)	3 (3.3)	3 (3.3)	0 (0.0)	23 (25.6)	15 (16.7)	26 (28.9)	90 (32.1)
Overall apron (yes)	19 (9.3)	10 (4.9)	12 (5.9)	3 (1.5)	2 (1.0)	62 (30.4)	30 (14.7)	66 (32.4)	204 (72.9)

n (%) represent frequency and row percentage. JHS (Junior High School), MSLC (Middle School Leaving Certificate

The waste handlers worked with different waste management organisations, anonymised, including; PC_1 (n = 155; 55.4%): Private Company 1 (workers in both Lower and Upper communities–waste handlers worked at the beaches, in open drains, cemeteries, streets), AC (n = 20; 7.1%): Area Council (workers in both Lower and Upper communities–waste handlers worked at public toilet facilities), PC_2 (n = 9; 3.2%): Private Company 2 (workers at Upper communities–waste handlers worked in open drains, cemeteries, streets), PC_3 (n = 51; 18.2%): Private company 3 (workers at Lower communities–waste handlers worked at the beaches) and CVs (n = 45; 16.1%): Community Volunteers (workers at Upper communities–waste handlers worked on the streets).

The proportions of waste handlers performing different activities were as follows: sweeping only (n = 51; 18.2%), disposal only (n = 18; 6.4%), collection only (n = 12; 4.3%) and transport only (n = 5; 1.8%). Workers who performed two or more activities included; sweeping, collection and disposal (n = 83; 30.0%), sweeping and collection (n = 69; 25.0%), collection and disposal (n = 36; 12.9%) as well as sweeping and disposal (n = 6; 2.1%).

The overall distribution of use and type of PPE during work was as follows: Wellington boots (n = 173; 61.8%), gloves (n = 161; 57.5%), nose/mouth cover (n = 90; 32.1%) and overall apron (n = 204; 72.9%) (Table 1). The proportion of waste handlers with the highest use for gloves (n = 51; 32%) were those who performed sweeping, collection and disposal activities, whilst none of the transporters used gloves during work. Overall, about one-fifth (n = 59; 21.1%) of the participants did not use any type of PPE during work while (n = 221, 78.9%) used at least one type of PPE.

### Prevalence and concentration of *E*. *coli*

There were no visible growth of colonies on agar plates (no detection of *E*. *coli*) for the negative controls. None of the before work hand-rinse samples yielded too numerous to count colonies whilst about 4% (n = 12) of the after work samples initially yielded too many colonies and therefore required testing the next day after 10^−1^ serial dilution. The prevalence of *E*. *coli* on hands of waste handlers engaged in the different types of activities before and after work were 5.0% (95% CI: 3.3%-7.1%) and 23.2% (95% CI: 18.4%-28.6%), respectively. Waste handlers with the following characteristics had lower prevalence of *E*. *coli* detected after work; females (n = 41; 19.4%), waste handlers below 35 years (n = 9; 10.0%), those with secondary level education (n = 2; 15.4%), those who earned less than 80 GH₵ (< USD 36) a month (n = 54; 23.0%) and those who have worked a minimum of two years with waste management organisation (n = 6; 19.0%).

Using a paired t-test, the log concentration of *E*. *coli* on hands of workers engaged in the different types of activities after completing a day’s work (8.60 ± 4.20 CFU/hand, mean ± standard deviation) was found to be significantly higher compared with the log concentration before the initiation of work (2.95 ± 1.89 CFU/hand, mean ± standard deviation) (p < 0.001). The differences in the average log of *E*. *coli* concentration among workers engaged in the different types of waste handling activities was, however, not statistically significant (p = 0.105; Table 2). The detection of *E*. *coli* on hands of the waste handlers was also not dependent on the use of PPE, based on the Welch t-test (p > 0.05, Table 3).

**Table 2:** Distribution of mean log concentrations of *E*. *coli* among waste handlers performing different activities.

Waste handling activities	n	Mean ± SD	P-value
Sweeping	51	5.32 ± 3.77	
Disposal	18	7.72 ± 5.60	
Collection	12	4.81 ± 3.10	
Transport	5	5.96 ± 4.58	
Sweep & Disposal	6	3.91 ± 0.01	
Sweeping & Collection	69	5.41 ± 3.73	
Disposal & Collection	36	6.85 ± 4.65	
Sweeping & Collection & Disposal	83	5.37 ± 3.15	
Overall	280	5.67 ± 3.85	0.105

Mean log difference: Row mean log–Column mean log. P-values from paired t-test, unit of measurement for mean log concentrations of E. coli was CFU/hand.

**Table 3:** Distribution of mean log concentrations of *E*. *coli* among waste handlers by use of personal safety working gears and exposure surfaces.

		No	Yes	P-value
	n	(Mean ± SD)	(Mean ± SD)	
Type of safety working gear				
Wellington Boot	280	5.53 ± 3.93	5.72 ± 3.82	0.692
Glove	280	5.60 ± 3.96	5.68 ± 3.79	0.873
Mouth/nose cover	280	5.58 ± 3.81	5.79 ± 3.96	0.675
Overall apron	280	5.81 ± 4.24	5.59 ± 3.71	0.688
Exposure surfaces				
Mouth and nose	280	5.82 ± 3.94	5.41 ± 3.73	0.375
Hands	280	5.23 ± 3.32	5.72 ± 3.94	0.407
Legs/feet	280	5.64 ± 3.86	5.81 ± 3.80	0.935

SD: Standard deviation, p-value obtained from Welch t-test, unit of measurement for mean log concentrations of E. coli was CFU/hand, n = frequency.

### Risk factors associated with detection of *E*. *coli* on hands of waste handlers

The multivariable analysis of factors associated with faecal hand pollution showed that age, sex and working with the waste management organisation (AC) were significantly related to detection of *E*. *coli*. The odds of detecting *E*. *coli* was significantly higher (aOR 4.2; 95% CI: 1.9–9.1) among waste handlers aged 35 years and above compared to those less than 35 years, controlling for type of waste management organization and sex. The odds of detecting *E*. *coli* for waste handlers who worked with the organization AC (at public toilet facilities) was three times (aOR 3.0; 95% CI: 1.0–8.4) higher compared with workers employed by the organisation PC_1 when controlling for age and sex (Table 4). Female waste handlers were found to be 60% (aOR 0.4; 95% CI: 0.2–0.8) less likely to experience faecal pollution compared with males workers, controlling for age and type of waste management organisation (Table 4).

**Table 4:** The effect of socio-demographic factors and other determinants on detection of faecal indicator *E*. *coli*.

	Unadjusted			Adjusted	
	n (%)	OR (95%CI)	P-value	OR (95%CI)	P-value
**Sex**			0.01		<0.009
Male	69 (24.6)	1		1	
Female	211 (75.4)	0.5 (0.2–0.8)		0.4 (0.2–0.8)	
**Age in years**			0.001		<0.0001
<35	90 (32.1)	1		1	
35 and above	190 (67.9)	3.8 (1.8–8.0)		4.2 (1.9–9.1)	
**No. of Years worked**			0.798		****
< 1 year	162 (57.9)	1		****	
1–2 years	32 (11.4)	0.7 (0.3–1.8)		****	
3–4 years	77 (27.5)	0.8 (0.4–1.5)		****	
5 or more years	9 (3.2)	0.8 (0.2–4.2)		****	
Highest Education Level			0.348		
None	24 (21.0)	1		****	
Primary	16 (20.5)	1.0 (0.5–2.0)		****	
JHS	10 (33.3)	1.9 (0.8–4.6)		****	
MSLC	13 (30.0)	1.6 (0.7–3.5)		****	
Secondary	2 (15.4)	0.7 (0.1–3.3)		****	
Current Monthly Salary			0.690		
Less than ¢80.0 (< USD 36)	11 (26.0)	1		****	
¢80-¢150 (USD 36–45)	54 (23.0)	0.9 (0.4–1.8)		****	
**Waste Handling Activities**			0.483		****
Sweeping	8 (16.0)	1		****	
Disposal	3 (17.0)	1.1 (0.3–4.6)		****	
Collection	5 (42.0)	3.8 (1.0–15.2)		****	
Transportation	3 (60.0)	8.1 (1.2–56.2)		****	
Sweeping and Disposal	2 (33.3)	2.7 (0.4–17.2)		****	
Sweeping and Collection	15 (21.7)	1.5 (0.6–3.8)		****	
Collection and Disposal	11 (31.0)	2.4 (0.8–6.7)		****	
Sweeping, Collection and Disposal	18 (22.0)	1.5 (0.6–3.7)		****	
**Waste handling Organisation**			0.038		<0.009
PC_1	39 (25.2)	1		1	
AC	10 (50.0)	3.0 (1.1–8.0)		3.0 (1.0–8.4)	
PC_2	1 (11.1)	0.4 (0.1–3.1)		0.4 (0.0–3.0)	
PC_3	10 (19.6)	0.7 (0.3–2.0)		0.6 (0.3–1.4)	
CVs	5 (11.1)	0.4 (0.1–1.0)		0.3 (0.1–1.0)	
**Type of safety working gear**					
Wellington boot	38 (22.0)	0.8 (0.5–1.5)	0.529	****	****
Gloves	37 (23.0)	1.0 (0.6–1.7)	0.914	****	****
Mouth/nose cover	21 (23.3)	1.0 (0.6–1.8)	0.974	****	****
Overall apron	45 (22.1)	0.8 (0.4–1.5)	0.454	****	****
**Exposure surfaces**					
Mouth and nose	28 (23.9)	1.1 (0.6–1.9)	0.81	****	****
Hands	55 (23.0)	0.9 (0.4–2.0)	0.847	****	****
Leg/feet	32 (25.0)	1.2 (0.7–2.1)	0.516	****	****

n (%) represent frequency and row percentage / prevalence of E. coli. JHS (Junior High School), MSLC (Middle School Leaving Certificate

**** Represent covariates that were not associated with faecal contamination at the univariable analysis stage and therefore no parameter estimation at the multivariable stage. OR is unadjusted odds ratio. Adjusted OR is adjusted odds ratio estimate from a multivariable binary logistic regression analysis. PC_1: Private Company 1(workers in both Lower and Upper communities–waste handlers worked at the beaches, in open drains, cemeteries, streets). AC: Area Council (workers in both Lower and Upper communities–waste handlers worked at public toilet facilities). PC_2: Private Company 2 (workers at Upper communities–waste handlers worked in open drains, cemeteries, streets). PC_3: Private company 3 (workers at Lower communities–waste handlers worked at the beaches). CVs: Community Volunteers (workers at Upper communities–waste handlers worked on the streets)P-values were derived from logistic regression model.

## Discussion

The observation of defecation activities by the residents of the study area in open spaces as well as the physical presence of faecal material in solid waste is consistent with findings of a baseline survey conducted in Prampram, by the Sustainable Sanitation (SUSA) project (SUSA, 2011) [31] in which most of the households (75%) did not have their own toilet facilities, with a majority (76%) of residents practicing open defecation [31]. Spencer (2012) [18] also found that over 50% of the residents of were not satisfied with their present environmental conditions. Waste handlers were thus seemed to be exposed to the hazards of faecal pollution of the solid waste generated from within the communities.

In this study, we report significantly higher proportion of female engagement in sweeping compared with males (Table 1). This finding corroborates previous studies conducted by Agwu (2012) [32] in Nigeria and Kadfak (2011) [33] in Ghana. There is a traditional perception in most African communities that waste handling activities like cleaning and sweeping, are predominantly female jobs. Among certain minority tribes in Nigeria, it is believed that a male is likely to lose sexual potency when touched by locally prepared brooms, used for sweeping [34].

We found that female waste handlers were less likely to experience faecal pollution of their hands compared with male workers (Table 4). Thus female waste handlers might have adopted better protective behaviour at work by using PPE and adopting personal hygiene practices. Even though waste handlers were aware of the importance and timing of hand hygiene practices and use of sanitation facilities, observation at the work sites revealed that access to convenient hand hygiene facilities (water and soap for washing hands after work, after defecation and before eating) was lacking.

We also found that a majority of waste handlers had no formal education and had a low average monthly income (Table 1). Indeed the observation from transect walk of the peri-urban community showed that waste handlers used rudimentary waste handling equipment and technology, which did not require high level of education to operate. The use of such equipment, however, may compromise efficiency of work plus either induce or aggravate already existing health problems among workers [5, 35]. On the other hand, the lack of formal education could predispose waste handlers to the hazards of work since they may have limited understanding about appropriate safety procedures to adopt during work [36]. Waste handlers in LMICs constitute one of the least paid workers regardless of the reported high levels of health risks [11]. The findings of this study showed that the highest income earned through waste handling was 150 GH₵ (approx. USD 29) a month, which was the main source of income for most workers. This was however, far lower than the average monthly per capita income in Ghana, i.e. 225 GH₵ (approx. USD 44) (GSS, 2013).

Unlike the findings of Dodrill *et al*., (2011), [37] in which younger workers were more likely to be at risk of faecal pollution of hands, our study found rather that older workers aged 35 years and above had higher likelihood of experiencing faecal pollution of their hands compared with younger waste handlers. This effect may be due to the increasing reluctance in adopting safety measures among older workers compared with the younger waste handlers, presumably because of apathy or other differences in lifestyle and personal behaviour, not captured by this study.

The protection of waste handlers from the hazards of work could be achieved by measures such as adopting safety procedures, having access to appropriate sanitation and hygiene facilities or by using PPE during work [38]. Even though the use of PPE are strongly recommended to prevent injuries, workplace hazards and diseases that are likely to arise from the working environment [39], about half the number of waste handlers did not use them regularly. From the transect walk and observation, non-compliance to the use of PPE, in spite of having access to it may be linked to the discomfort in its use during a workday, considering the strenuous activities under hot and humid climatic conditions or other reasons not directly captured in this study [40]. Even though the use of PPE does not remove the source of health hazards in waste handling, it can provide benefits that outweigh the harm and personal discomfort associated with them and through proper use, can contribute to health and safety of workers [41].

Our findings suggest that compared with those who were employed by the waste management organisation PC_1, waste handlers who worked for the AC had greater likelihood of having faecally polluted hands (Table 4). Factors including the longer duration of work at the public toilet facilities and unsuitable utilization of PPE among the workers of AC might explain this finding. Waste handlers employed by PC_1 had spent less than a year with handling waste, they did not work at public toilet facilities and used relatively newer and supervised PPE, compared with workers of the AC waste management organisation. Public health efforts by the employers of waste handlers, at reducing faecal pollution at work, must most importantly target the workers of AC.

Our study reported significantly higher concentration of faecal pollution after completing a day’s waste handling activities, a finding which suggests faecal exposure in the solid waste-stream being handled. Literature relating our findings of faecal pollution on hands of waste handlers with previous empirical findings in other LMICs was limited. However, studies from related sites in Tanzania [30], Vietnam [42], Bangladesh [43] as well as Ghana [44] reported higher levels of faecal pollution of the hands after toilet cleaning activities, which were comparable to our findings. Combined, these findings might imply that the direct contact of the bare hands to bacteria and possible pathogens in faecal polluted solid waste, due to widespread open defecation in poor coastal peri-urban communities, poses serious health risks to waste handlers working in such environments [3, 45–47]. The presence of pathogens on hands due to faecal pollution, can serve as transmission pathways to gastrointestinal tract problems like typhoid fever and diarrhoea as also reported from poor peri-urban settings in Malaysia [48], and in other parts of Africa [12, 13, 38, 49].

Despite that a majority of waste handlers (almost 80%) had no *E*. *coli* detected on their hands after work, a significant increase in faecal pollution of hands was still seen, thus correct use of PPE should be promoted to ensure health and safety measures to avoid faecal pollution among the workers. Therefore, waste management organisations employing large number of waste handlers in resource-deprived peri-urban settings in Southern Ghana should provide and supervise the use of adequate hygiene facilities (soap and water) during waste handling. There is also the need to draw attention to waste management organisations about their responsibility to provide, instruct and train in correct usage of PPE to effectively protect the health of workers.

### Limitations

This study acknowledges the limitation that a comparative study group to serve as controls was not used to ascertain faecal pollution of hands. Also, the rather modest sample size (n = 65) of workers with faecal pollution on hands used for the analysis of associated risk factors may affect the statistical power of the study. It would have been interesting also to see whether presence of *E*. *coli* correlates with concentrations of other microorganisms, including pathogens on hands. Despite these limitations, the study has provided new important information on the extent and risk factors to faecal pollution of hands among waste handlers in a resource-poor community in a LMIC country.

## Conclusion

The findings from this study suggest that waste handlers in resource-deprived peri-urban settlements in LMICs are exposed to the hazards of faecal pollution of solid waste, with the male workers and those above 35 years being the most vulnerable group to experience faecal pollution of the hands. Efforts by local waste management organisations at reducing exposures to faecal pollution to waste handlers must include improvement in the provision and supervised use of sanitation/hygiene facilities and PPE Residents in the local communities must be aware of the dangers of open defecation and avoid the practice. Whilst the detection of faecal indicator *E*. *coli* would not direct be a cause of disease, its presence indicates that faecal pathogens may also be present. Thus, future studies to identify potential faecal-associated human pathogens, and antimicrobial susceptibility of those of bacterial origin among the workers, are recommended.

## Supporting information

S1 Dataset (DTA)

## Abbreviations

ANOVA: Analysis Of Variance
aOR: Adjusted Odds Ratio
CFU/ml: Colony Forming Units per millilitre
*E*. *coli*: *Escherichia coli*
GSS: Ghana Statistical Services
PPE: Personal Protective Equipment
SUSA: Sustainable Sanitation
TPC: Total Plate Count
